# Advances in electroacupuncture for perioperative neurocognitive disorders: mechanisms and clinical evidence

**DOI:** 10.1186/s13020-026-01466-1

**Published:** 2026-07-08

**Authors:** Shirong Wei, Sitong Zhou, Junwen Tu, Tong Zhi, Yungong Wang, Qihong Shen, Chaobo Ni, Ming Yao, Huadong Ni

**Affiliations:** 1 Zhejiang Chinese Medical University, Hangzhou, 310053 China; 2 Department of Anesthesiology and Pain Research Center, The Affiliated Hospital of Jiaxing University, No. 1882 South Zhonghuan Road, Jiaxing, 314001 China

**Keywords:** Electroacupuncture, Perioperative neurocognitive disorders, Postoperative delirium, Postoperative cognitive dysfunction

## Abstract

Perioperative neurocognitive disorders (PND), including postoperative delirium, delayed neurocognitive recovery, and postoperative cognitive dysfunction, are common complications in older surgical patients and are associated with impaired recovery, reduced quality of life, and increased postoperative morbidity. Current management remains largely supportive and preventive, and effective targeted therapies are still lacking. Electroacupuncture (EA), as a minimally invasive neuromodulatory intervention, has attracted increasing attention because of its potential multi-target regulatory effects. This review summarizes current mechanistic and clinical evidence regarding EA for PND. Preclinical studies suggest that EA may modulate several interacting pathological processes, including neuroinflammation, oxidative stress, autophagy dysfunction, ferroptosis, mitochondrial injury, microbiota-gut-brain axis dysregulation, and hippocampal synaptic plasticity. Recent PND/POCD animal studies further support EA-related regulation of NLRP3 inflammasome activation, cGAS–STING signaling, SIRT1/NRF2/GPX4-mediated ferroptosis, AMPK/SIRT1/FOXO1/PINK1/Parkin-related autophagy pathways, and MAPK-related synaptic plasticity. Clinical studies and meta-analyses suggest that EA and related acupoint-based electrical stimulation techniques may reduce early postoperative cognitive decline and improve short-term cognitive outcomes in older surgical patients. However, the overall evidence remains limited by heterogeneous stimulation protocols, variable acupoint prescriptions, incomplete blinding, short follow-up, and reliance on cognitive screening scales. Several proposed mechanisms are still partly inferred from non-PND models. Future studies should use standardized EA protocols, clinically relevant PND models, dynamic mechanistic assessments, and adequately powered sham-controlled trials to clarify the therapeutic role of EA in PND.

## Introduction

With the global aging of the population, the increasing number of elderly patients undergoing surgery has made perioperative neurocognitive disorders (PND) a significant clinical and public health concern [1]. In 2018, a consensus nomenclature defined PND as an umbrella term for cognitive changes related to surgery and anesthesia [2]. This broad term encompasses a spectrum of clinical states, including postoperative delirium (POD), a potentially reversible, acute-phase cognitive dysfunction characterized by deficits in attention and cognitive processing [3], delayed neurocognitive recovery (dNCR), and postoperative neurocognitive disorder (POCD), which may persist for months or longer [4]. The incidence of PND is high and varies significantly by surgical type and patient population. For example, POD rates range from 16.4 to 50% in cardiac surgery patients, while the incidence of POCD at 3 months post-surgery in older non-cardiac patients is between 9.9 and 12.7% [5]. The occurrence of PND not only severely impairs patients' quality of life but also serves as an independent predictor of increased postoperative mortality. Although its pathophysiology is multifactorial—involving neuroinflammation, neuronal injury, hypercoagulability, anesthetic neurotoxicity, and EEG burst suppression—neuroinflammation is widely considered an important contributor to PND [6].

Despite increasing recognition of PND, current management remains largely supportive and preventive, and no specific therapy has been conclusively established. Existing pharmacological interventions and perioperative optimization strategies have shown variable efficacy across studies, and some may be limited by adverse effects, narrow therapeutic targets, or poor applicability in frail older surgical patients. Moreover, because PND is driven by multiple interconnected mechanisms rather than a single pathogenic pathway, conventional single-target interventions may be insufficient to address the complex pathophysiology of PND. Therefore, there is a strong rationale for exploring non-pharmacological approaches with multimodal regulatory effects.

Electroacupuncture (EA) is a minimally invasive and clinically applicable neuromodulation technique that applies low-intensity electrical currents to specific neuroanatomical sites (acupoints) to stimulate peripheral nerves, thereby modulating central nervous system function [7]. Previous studies suggest that EA may modulate neuroinflammation and related neuroprotective pathways. Because PND shares several pathological features with chronic neurodegenerative conditions, such as neuroinflammation and synaptic dysfunction, evidence from Alzheimer’s disease, Parkinson’s disease, and related cognitive disorders provides an indirect rationale for exploring EA in PND [8]. However, the efficacy and disease-specific mechanisms of EA in PND still require direct validation.

This review summarizes current evidence on the mechanisms and clinical value of EA for PND. We focus on recent progress in modulating key pathological pathways, such as neuroinflammation, oxidative stress, and autophagy [9], as well as emerging evidence related to apoptosis, mitochondrial energy metabolism, and omics-based molecular profiling. Additionally, we assess current clinical evidence to develop a cautious “bench-to-bedside” perspective on the potential translational application of EA in PND prevention and management.

## The mechanism of EA in PND

The pathophysiology of PND is complex and multifactorial, involving interlinked processes such as neuroinflammation, oxidative stress, autophagy dysfunction, synaptic impairment, mitochondrial injury, and other forms of cellular stress. Surgical trauma and anesthesia may initiate these processes through systemic inflammation, blood–brain barrier disruption, neuroimmune signaling, pain- and stress-related responses, and gut–brain axis dysregulation. Within the CNS, these processes may converge on neuronal injury, impaired synaptic plasticity, altered neural activity, and network dysfunction, ultimately contributing to postoperative cognitive impairment. This section discusses the roles of these mechanisms in PND and summarizes how EA may modulate these pathological processes. To help readers compare the available preclinical evidence, we summarized key information from EA studies conducted in PND or postoperative cognitive impairment models. As detailed in Table 1, this summary includes the specific animal models, acupoint selection, EA stimulation parameters, tissue sampling sites, and the key signaling pathways and molecular targets implicated in the potential effects of EA.

**Table 1:** Summary of direct EA studies in PND animal models

References	Model	Acupoint	EA parameter	Sample site	Pathways	Main findings	Model/Disease context
Feng et al. [36]	Hepatectomy rat	GV20, GV14	1 mA; 15 Hz (square); 30 min; 5 days	Hippocampus	–	IL-1β, IL-6, TNF-α, HMGB-1,TLR 4/2↓	PND model
Zhou et al. [43]	Tibial fracture rat	GV20	1 mA; 2/15 Hz; 30 min; 5 days	Blood, hippocampus	α 7nAChR	IL-1β, HMGB-1, TNF-α, mast cell, apoptosis↓	PND model
Sun et al. [54]	Hepatectomy mice	GV20	0.5 mA; 2 Hz; 20 min; twice daily; 7 days	Hippocampus	NF-κB, NLRP3 inflammasome,	Microglial, IL-1β, IL-6↓	PND model
Ma et al. [56]	Splenectomy rat	GV20, PC6, ST36	1 mA; 2/15 Hz (sparse-dense); 30 min; 3 days	Hippocampus	cGAS–STING, NF-κB, IRF3	cGAS, STING, NF-κB p65, IRF3, IL-1β↓	PND model
Liu et al. [62]	Hepatic lobe resection rat	GV20, PC6, LI4	4 mA; 2/100 Hz (continuous); 30 min; 8 days	Hippocampus, blood	–	GFAP, S-100β, NSE, MDA↓ BDNF, GDNF, SOD↑	PND model
Zhu et al. [65]	Appendectomy mice	ST36, LI11, GV20, GV14	–; 15 Hz (continuous); 30 min; 11 days	Hippocampus, fecal	Gut microbiota	PDGF↑ Helicobacteraceae, Actinomycetes, Clostridium sensu stricto 1, Escherichia/Shigella, GFAP, DAO, LPS, TNF-α, IL-1, IL-6↓	PND model
Chen et al. [83]	Tibial fracture mice	GV20	1 mA; 20 Hz (sparse wave); 30 min; 5 days	Hippocampus	TFR1-DMT1-FPN axis	GSH, FPN, GRX1, p-GSK-3β, Nrf2, HO-1 ↑ Ferroptosis, MDA, Fe, TFR1, DMT1 ↓	PND model
Zhang et al. [84]	Hepatectomy mice	GV20	0.5 mA; 2 Hz; 20 min; 7 days; twice a day	Hippocampus	GRX1/GSK-3β/Nrf2 axis	GRX1, p-GSK-3β, Nrf2, HO-1, GPX4, SLC7A11, FTH1, GSH ↑ Ferroptosis, MDA, Fe, TFR1, DMT1 ↓	PND model
Du et al. [132]	Tibial fracture mice	GV20, GV14	1 mA; 2/15 Hz (sparse-dense); 30 min; 5 days (pretreatment)	Hippocampus	SIRT1/NRF2/GPX4	SIRT1, NRF2, GPX4, SLC7A11, ATP and mitochondrial membrane potential↑; Fe, ferroptosis↓	PND model
Cheng et al. [125]	Laparotomy rat	PC6, LI4, ST36	2/15 Hz, 1 mA, 5-day pretreatment	Hippocampal CA1	p38/MAPK	p-p38/p38↓; PSD-95 and synaptophysin↑; dendritic spine density and synaptic plasticity↑	PND model
Zhang et al. [88]	Abdominal rat	LI4, PC6, ST36	1 mA; 2/15 Hz (sparse-dense); −; 5 days	Hippocampus	Mitochondrial, Neuroapoptosis	Neuron, Ca2 +, Bcl-2↑ ROS, Cyt c, Bax, cleaved caspase 9, cleaved caspase 3, Bax/Bcl-2↓	PND model
Wang et al. [89]	Abdominal mice	GV20	1 mA; 2/15 Hz (disperse-dense); 10 min; 3 days	Hippocampal	–	Telomerase activity, TERT protein, SOD, LC3B-II/LC3B-I, Beclin-1↑ ROS, MDA, Iba1, IL-6, TNF-α↓	PND model
Niu et al. [110]	Laparotomy rat	GV14, GV20	1 mA; 3.85/6.25 Hz (sparse-dense); 30 min; 5 days	Hippocampus	AMPK	LC3II, Beclin-1, AMPK, p-AMPK, autophagic vesicles↓	PND model
Zhang et al. [111]	Abdominal mice	GV20, PC6	1 mA; 2/100 Hz (sparse-dense wave); 30 min; 5 days	Hippocampus	Autophagy pathway (SIRT1/FOXO1)	Beclin-1, SIRT1 SOD, CAT ↑ p62, FOXO1, Oxidative Stress ↓	PND model
Guo et al. [112]	Hepatic lobe resection rat	SP6, ST36	1.5 mA; 2–100 Hz (auto-shifting, sweeping); 30 min; 3 days	Hippocampus	Mitophagy pathway (PINK1/Parkin)	Mitophagy, PINK1, Parkin, LC3, Beclin1↑, IL-1β, NLRP3, ROS ↓	PND model
Huang et al. [124]	Abdominal rat	PC6, LI4, ST36	1 mA; 2/15 Hz (sparse-dense); −; 5 days	Hippocampus	ERK/MAPK/CREB	ERK/MAPK/CREB activation↑; synaptic plasticity-related signaling↑	PND model

### Neuroinflammation

Neuroinflammation is widely recognized as an important contributor to the onset and development of PND [10–13]. Perioperative stressors, such as surgical trauma and anesthesia, induce tissue injury and cell membrane damage, leading to the release of intracellular components [14–18]. These damaged cells release substantial amounts of damage-associated molecular patterns (DAMPs), including high mobility group box 1 (HMGB1), ATP, and uric acid, into the peripheral circulation [19, 20]. The release of these DAMPs represents an early step in the perioperative inflammatory response [21]. HMGB1 is a representative DAMP and late pro-inflammatory mediator that may link peripheral tissue injury with central neuroinflammatory activation. Although EA has been shown to reduce HMGB1-related neuroinflammation in sepsis-associated encephalopathy, this evidence remains indirect for PND [22]. Therefore, HMGB1-related signaling may be a biologically plausible EA-related mechanism in PND, but it still requires direct validation in PND-specific models.

In addition to triggering peripheral inflammation, DAMPs and associated pro-inflammatory cytokines may disrupt blood–brain barrier (BBB) integrity, thereby promoting the entry of circulating inflammatory mediators and immune cells into the central nervous system [18, 23–25]. This process may involve endothelial dysfunction, downregulation of tight junction proteins such as claudin-5, occludin, and ZO-1, and increased matrix metalloproteinase activity, ultimately amplifying central neuroinflammatory responses and neuronal dysfunction [26]. Evidence from related neurological conditions suggests that EA may influence BBB permeability in a context-dependent manner. Some studies indicate that EA can transiently increase BBB permeability within a therapeutic window, which may facilitate targeted drug delivery, whereas other studies suggest that EA may help preserve BBB integrity by reducing pro-permeability factors such as MMP-9 and VEGF and by maintaining tight junction proteins [27]. Potential signaling mechanisms may involve Shh-Gli1- and NMDA receptor-related pathways [28–30]. However, most evidence for EA-mediated BBB regulation is derived from non-PND models, and direct validation in perioperative or EA-treated PND models remains limited. Therefore, BBB regulation should be regarded as a plausible but not yet fully established EA-related mechanism in postoperative neuroinflammation.

Increased BBB permeability may allow peripheral inflammatory mediators and DAMPs to enter the CNS, where they can activate pattern recognition receptors such as Toll-like receptors (TLR2 and TLR4) and the receptor for advanced glycation end products (RAGE) [24, 31–35]. In a postoperative cognitive impairment model, EA at Baihui (GV20) and Dazhui (GV14) attenuated postoperative neuroinflammation and cognitive impairment in aged rats by downregulating hippocampal TLR2 and TLR4 expression, inhibiting microglial activation, and reducing the release of pro-inflammatory cytokines, including IL-1β, IL-6, TNF-α, and HMGB1 [36]. This study provides direct PND-model evidence that EA may regulate TLR-related inflammatory signaling. By contrast, evidence for EA-mediated regulation of the HMGB1/RAGE pathway is mainly derived from cerebral ischemia models, in which EA at GV20 and ST36 suppressed HMGB1 and RAGE expression and reduced neuroinflammation and neuronal injury [37, 38]. Therefore, DAMPs–RAGE/HMGB1 signaling should currently be regarded as an indirect but biologically plausible EA-related mechanism in PND that requires further validation in postoperative models. In addition to resident glial cells, peripheral immune cells may further amplify neuroinflammation after CNS entry or activation. Neutrophils can release pro-inflammatory cytokines, reactive oxygen species, and matrix metalloproteinases, thereby aggravating BBB injury and neuronal damage [39, 40]. Mast cells (MCs) may also contribute to neuroinflammation by releasing histamine, cytokines, and other mediators, increasing BBB permeability and promoting microglial and astrocytic activation [41, 42]. In a tibial fracture-induced postoperative cognitive impairment model, EA reduced hippocampal MC degranulation and alleviated neuroinflammation and cognitive impairment through α7 nicotinic acetylcholine receptor (α7nAChR)-related signaling [43]. These findings suggest that modulation of MC activation may represent another PND-relevant anti-inflammatory mechanism of EA, although further validation is still needed.

After DAMPs activate pattern recognition receptors, intracellular inflammatory cascades such as the MyD88-dependent and TRIF-dependent pathways can be initiated. These pathways promote the activation of transcription factors, including NF-κB and MAPK, which regulate the expression of pro-inflammatory cytokines and inflammasome-related genes [44–46]. Evidence from traumatic brain injury and vascular dementia models suggests that acupuncture at acupoints such as Baihui (GV20) may attenuate neuroinflammation by inhibiting TLR4/MyD88-related signaling, but this evidence remains indirect for PND [47, 48]. Among downstream inflammatory mediators, the NLRP3 inflammasome is particularly relevant because it promotes the maturation and release of IL-1β and IL-18, thereby amplifying central neuroinflammation [49–53]. Direct PND-model evidence indicates that EA at ST36 and GV20 can inhibit hippocampal NLRP3 inflammasome activation, reduce microglial activation, modulate the M1/M2 polarization balance, and improve postoperative cognitive impairment [54]. Mechanistically, EA appears to inhibit both the priming and activation phases of the NLRP3 inflammasome by downregulating NLRP3, apoptosis-associated speck-like protein containing a CARD (ASC), and Caspase-1, thereby reducing IL-1β and IL-18 release. Additional support from Alzheimer’s disease models suggests that NLRP3-related regulation may be a broader anti-inflammatory mechanism of EA [55]. Overall, current evidence supports NLRP3 inflammasome regulation as one of the more directly supported EA-related inflammatory mechanisms in PND, although further validation across different surgical models is still needed.

More recently, Ma et al. provided direct evidence from an EA-treated PND animal model for cGAS–STING-related neuroinflammatory regulation [56]. In an aged rat model of sevoflurane-plus-splenectomy-induced postoperative cognitive dysfunction, EA at GV20, PC6, and ST36 improved postoperative cognitive deficits and reduced hippocampal cGAS–STING pathway activation, together with downstream NF-κB/IRF3-related inflammatory signaling. Immunofluorescence analysis further suggested that cGAS and STING activation was mainly localized in hippocampal neurons. This study suggests that cGAS–STING signaling is a PND-relevant inflammatory pathway that may be modulated by EA.

Pro-inflammatory cytokines can further amplify neuroinflammation by activating glial-cell signaling pathways such as NF-κB, MAPK, and JAK/STAT, thereby promoting microglial polarization toward the pro-inflammatory M1 phenotype and enhancing astrocytic reactivity [57–60]. One plausible mechanism by which EA may ameliorate PND is through regulation of glial activation states [61]. Liu et al. found that EA at Baihui (GV20), Neiguan (PC6), and Hegu (LI4) reduced GFAP-positive astrocytic activation, suppressed microglial activation, and shifted microglial polarization from the pro-inflammatory M1 phenotype toward the anti-inflammatory M2 phenotype in a POCD animal model [62]. These changes were accompanied by reduced IL-1β and TNF-α levels, lower oxidative stress, and improved cognitive function. Thus, modulation of glial activation states, especially microglial M1/M2 balance, may represent an important anti-inflammatory mechanism of EA in POCD/PND models.

The microbiota–gut–brain axis is another pathway through which perioperative stress may amplify neuroinflammation. Surgery, anesthesia, opioids, and postoperative pain can induce gut dysbiosis and impair intestinal barrier integrity, allowing microbial products such as lipopolysaccharide (LPS) to enter the circulation and promote systemic and central inflammatory responses [63, 64]. Direct evidence from PND models suggests that EA may regulate this pathway. Zhu et al. reported that EA remodeled surgery-induced gut microbiota dysbiosis in a PND mouse model, with increased beneficial taxa such as Coprococcus and reduced potentially pathogenic taxa such as Helicobacteraceae; these changes were associated with improved intestinal and blood–brain barrier integrity and reduced hippocampal neuroinflammation [65]. Yang et al. further showed that surgical pain may act as an upstream trigger of gut dysbiosis, which contributes to microglial activation and synaptic pathology [66]. These findings collectively suggest that EA may attenuate postoperative neuroinflammation partly by modulating pain-related gut dysbiosis and downstream microbiota–gut–brain axis signaling.

**Fig. 1:**
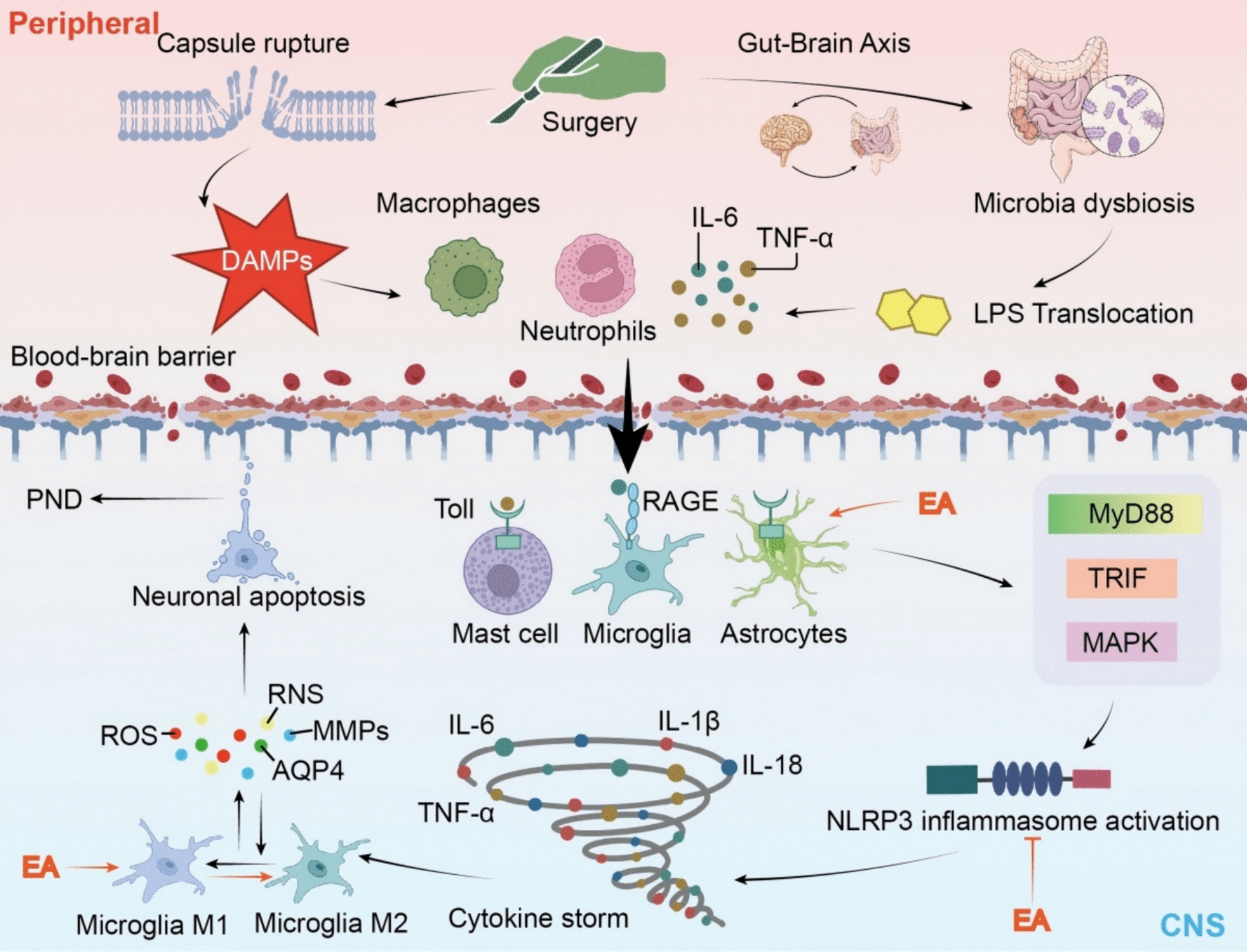
Mechanisms of neuroinflammation in PND and potential regulation by EA

Although current studies provide useful mechanistic clues for EA in modulating neuroinflammation and alleviating PND, several evidence gaps remain. Direct PND-model evidence is relatively stronger for NLRP3 inflammasome regulation, cGAS–STING-related neuroinflammatory signaling, mast cell activation, and microbiota–gut–brain axis modulation. In contrast, EA-mediated regulation of DAMPs–RAGE/HMGB1 signaling and BBB function is still supported mainly by studies in sepsis, cerebral ischemia, Alzheimer’s disease, or other related models, with limited direct validation in PND-specific settings. In addition, although the biphasic regulatory effect of EA on BBB permeability is theoretically relevant, its potential risks and clinical implications in the perioperative setting have not been fully evaluated. Future studies should prioritize EA-treated PND models and examine whether EA directly regulates BBB integrity, DAMPs-related signaling, and inflammatory cell activation in the perioperative brain. Overall, current evidence suggests that EA may have therapeutic potential for PND by modulating immune-cell activation, inflammasome signaling, cGAS–STING activation, and microbiota–gut–brain axis dysfunction. However, several of these mechanisms still rely partly on indirect evidence from non-PND models and require further validation in EA-treated PND models (Fig. 1).

Surgical trauma and anesthesia may induce peripheral tissue injury, release of damage-associated molecular patterns (DAMPs), activation of immune cells, gut microbiota dysbiosis, lipopolysaccharide (LPS) translocation, and blood–brain barrier (BBB) disruption. These peripheral events can promote the entry of inflammatory mediators into the central nervous system and activate Toll-like receptor-, RAGE-, MyD88/TRIF-, MAPK-, and NLRP3 inflammasome-related signaling pathways in microglia, astrocytes, mast cells, and other neuroimmune components. The resulting cytokine storm, glial activation, microglial M1/M2 imbalance, oxidative injury, and neuronal apoptosis may contribute to synaptic dysfunction and PND. EA may attenuate postoperative neuroinflammation by modulating immune-cell activation, glial polarization, inflammasome activation, gut–brain axis dysfunction, and inflammatory cytokine release. However, some pathways shown in this schematic, particularly HMGB1/RAGE-related signaling and BBB regulation, are supported partly by evidence from related non-PND models such as sepsis-associated encephalopathy, cerebral ischemia, or Alzheimer’s disease. Therefore, these pathways should be interpreted as indirect mechanistic support requiring further validation in EA-treated PND models.

### Oxidative stress

Oxidative stress refers to a pathological state in which the generation of reactive oxygen species (ROS) and reactive nitrogen species (RNS) exceeds endogenous antioxidant capacity, resulting in oxidative damage to lipids, proteins, nucleic acids, and organelles [67, 68]. In the perioperative setting, surgical trauma can promote ROS production through ischemia–reperfusion injury and systemic inflammatory responses, while anesthetic exposure may further impair mitochondrial function and antioxidant defenses [69–72]. These changes can damage neuronal structures, disturb synaptic function, and interact with neuroinflammatory pathways, thereby contributing to PND-related cognitive impairment [73, 74]. EA has therefore attracted attention as a potential multi-target intervention that may modulate oxidative stress-related pathways and support neuronal homeostasis.

Lipid peroxidation is a major form of oxidative injury in neurons because polyunsaturated fatty acids in cellular and mitochondrial membranes are highly vulnerable to ROS-mediated damage. Excessive lipid peroxidation can impair membrane fluidity, ion gradients, and signal transduction, increasing neuronal susceptibility to dysfunction and regulated cell death [75–77]. It is also a key driver of ferroptosis, an iron-dependent form of cell death characterized by lipid ROS accumulation [78–80]. Under perioperative stress, dysregulated iron metabolism, including increased iron influx and reduced iron export, may promote intracellular iron overload and Fenton reaction-mediated ROS production [81, 82]. PND-related studies suggest that EA may reduce ferroptosis-associated oxidative injury by regulating iron transport and antioxidant defense. For example, Chen et al. reported that EA downregulated iron influx proteins such as TFR1 and DMT1 and upregulated the iron efflux transporter FPN, thereby limiting iron overload [83]. Zhang et al. further showed that EA activated the GRX1/GSK-3β/Nrf2 pathway, promoted Nrf2 nuclear translocation, and increased downstream antioxidant and ferroptosis-inhibitory proteins such as GPX4 [84, 85]. These findings suggest that EA may reduce oxidative injury by coordinating iron homeostasis and Nrf2/GPX4-related antioxidant defense.

Mitochondria are both a major source and a major target of ROS. Surgery- and anesthesia-induced mitochondrial dysfunction can impair oxidative phosphorylation, reduce ATP production, disturb mitochondrial membrane potential, and promote ROS-induced ROS release, thereby amplifying oxidative injury [86, 87]. EA may protect mitochondrial function through several mechanisms. First, EA may attenuate mitochondria-mediated apoptosis by limiting calcium overload, stabilizing mitochondrial membrane potential, reducing mitochondrial permeability transition pore opening, and decreasing cytochrome c release and downstream caspase activation [88]. Second, EA pretreatment has been reported to promote mitochondrial translocation of telomerase reverse transcriptase (TERT), which may reduce mitochondrial ROS and contribute to neuroprotection [89]. In addition, EA-mediated regulation of mitophagy may help remove damaged mitochondria, as discussed in the autophagy section. These findings suggest that mitochondrial protection may be an important link between EA-mediated antioxidant effects and postoperative cognitive improvement, although the precise sequence of these events still requires further validation in PND models.

**Fig. 2:**
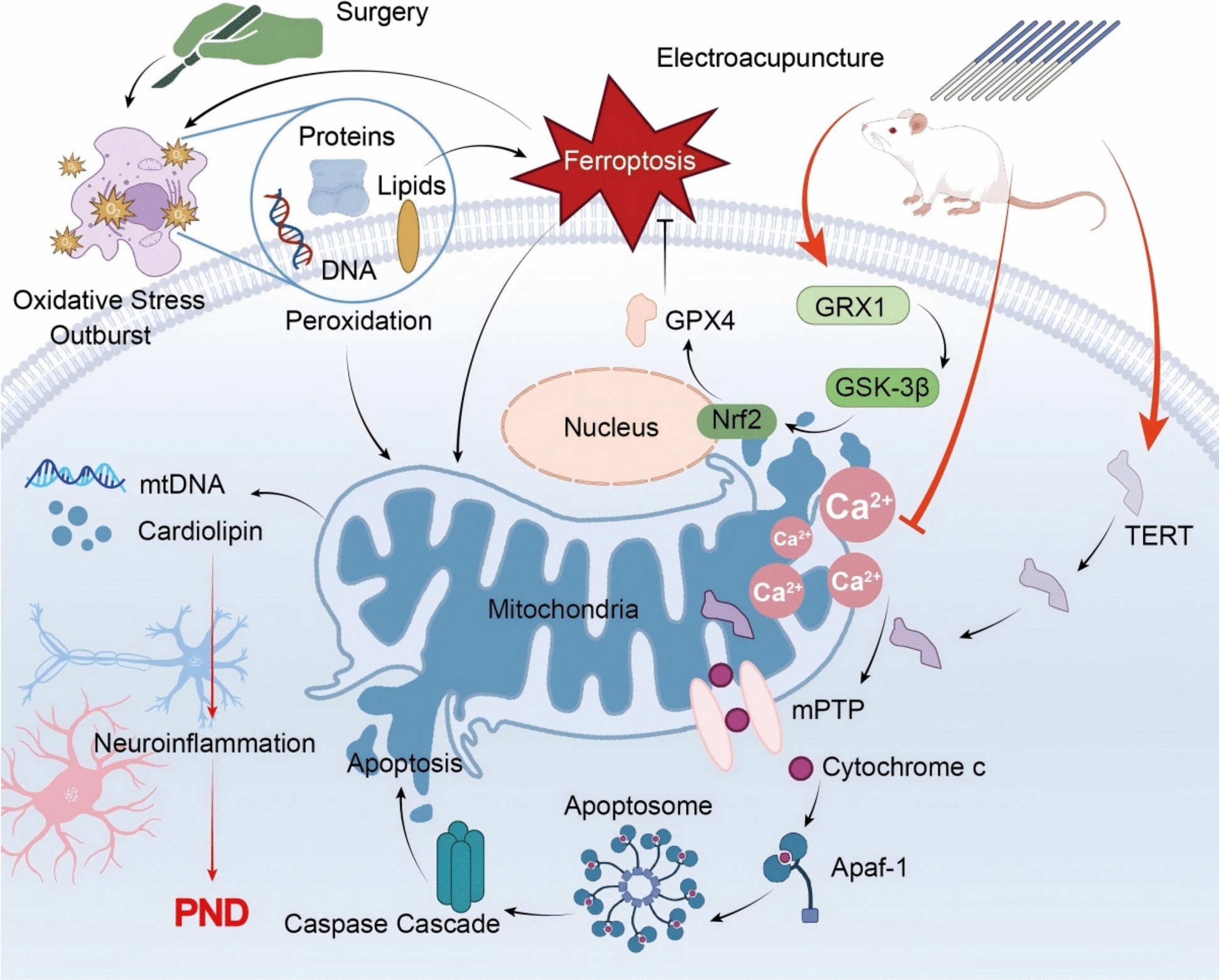
Mechanisms of oxidative stress in PND and potential regulation by EA

Oxidative stress and neuroinflammation can further reinforce each other. Damaged mitochondria may release mitochondria-derived DAMPs, such as mitochondrial DNA and cardiolipin, which activate innate immune pathways and promote microglial activation and pro-inflammatory mediator release [90–92]. Conversely, neuroinflammatory mediators can further impair mitochondrial function and increase ROS production. This reciprocal interaction may help explain how perioperative oxidative injury develops into sustained neuronal dysfunction and synaptic impairment. Overall, current evidence suggests that EA may modulate oxidative stress in PND by reducing lipid peroxidation and ferroptosis-related injury, enhancing Nrf2/GPX4 antioxidant signaling, preserving mitochondrial function, and limiting oxidative stress–neuroinflammation crosstalk. However, several mechanisms, especially TERT-mediated mitochondrial protection and mitochondria-derived DAMP signaling, still require direct validation in EA-treated PND models (Fig. 2).

Perioperative stress may induce oxidative stress through excessive reactive oxygen species production, lipid peroxidation, mitochondrial dysfunction, iron metabolic imbalance, and impaired antioxidant defense. These processes can damage proteins, lipids, and nucleic acids, promote ferroptosis, and trigger mitochondria-mediated apoptosis through calcium overload, mitochondrial permeability transition pore opening, cytochrome c release, Apaf-1 apoptosome formation, and caspase activation. EA may modulate these processes by regulating iron metabolism, activating GRX1/GSK-3β/Nrf2-related antioxidant signaling, maintaining GPX4-related ferroptosis defense, preserving mitochondrial membrane potential, promoting mitochondrial TERT translocation, and reducing apoptosis-related signaling. However, not all components shown in this schematic have been validated within a single unified PND model, and some mechanisms, such as TERT-mediated mitochondrial protection and mitochondria-derived DAMP signaling, still require direct validation in EA-treated PND models. Further studies are needed to clarify how these oxidative and mitochondrial pathways interact during EA-mediated neuroprotection in PND.

### Autophagy

Autophagy is a conserved intracellular degradation and recycling process that maintains cellular homeostasis by clearing damaged organelles, misfolded proteins, and abnormal protein aggregates [93]. In neurons, efficient autophagy is particularly important because long-lived cells depend on continuous protein quality control, mitochondrial turnover, and axonal transport. Dysregulated autophagy has been implicated in neurodegenerative diseases and PND, where impaired clearance of damaged mitochondria and toxic protein substrates may contribute to neuronal injury, synaptic dysfunction, and cognitive impairment [94–98].

In PND, autophagy dysfunction may arise from both abnormal upstream initiation and impaired downstream autophagic flux. Surgical trauma, anesthesia, oxidative stress, and neuroinflammation can disturb signaling pathways that regulate autophagy initiation, including mTOR-, AMPK-, and PI3K/Akt-related pathways [99–103]. At the same time, oxidative injury may impair lysosomal function and reduce degradative capacity, thereby blocking autophagic flux even when autophagosomes are formed [104–107]. This distinction is important because increased LC3-II or autophagosome accumulation may reflect either enhanced autophagy initiation or impaired downstream clearance. Dysfunctional autophagy can then promote the accumulation of p62, ubiquitinated proteins, damaged mitochondria, and immature autophagosomes, which may further aggravate oxidative stress, neuroinflammation, axonal transport defects, energy failure, and synaptic impairment [108, 109].

Available PND-model evidence suggests that EA may regulate autophagy through several signaling pathways. First, EA may modulate perioperative stress-induced abnormal autophagy initiation by regulating AMPK-related signaling and reducing excessive autophagosome accumulation [110]. Second, EA has been reported to activate the SIRT1/FOXO1 pathway, which may restore autophagic function by increasing Beclin-1 expression, reducing p62 accumulation, and improving neuronal ultrastructural integrity and cognitive performance [111]. Third, EA may enhance selective mitochondrial quality control by upregulating PINK1/Parkin-mediated mitophagy, thereby promoting the clearance of damaged mitochondria, improving mitochondrial structure and energy metabolism, reducing apoptosis-related injury, and alleviating spatial memory deficits [112]. These findings suggest that EA may not simply promote or inhibit autophagy in one direction; rather, it may help restore autophagic homeostasis by acting on different stages of the autophagy pathway.

**Fig. 3:**
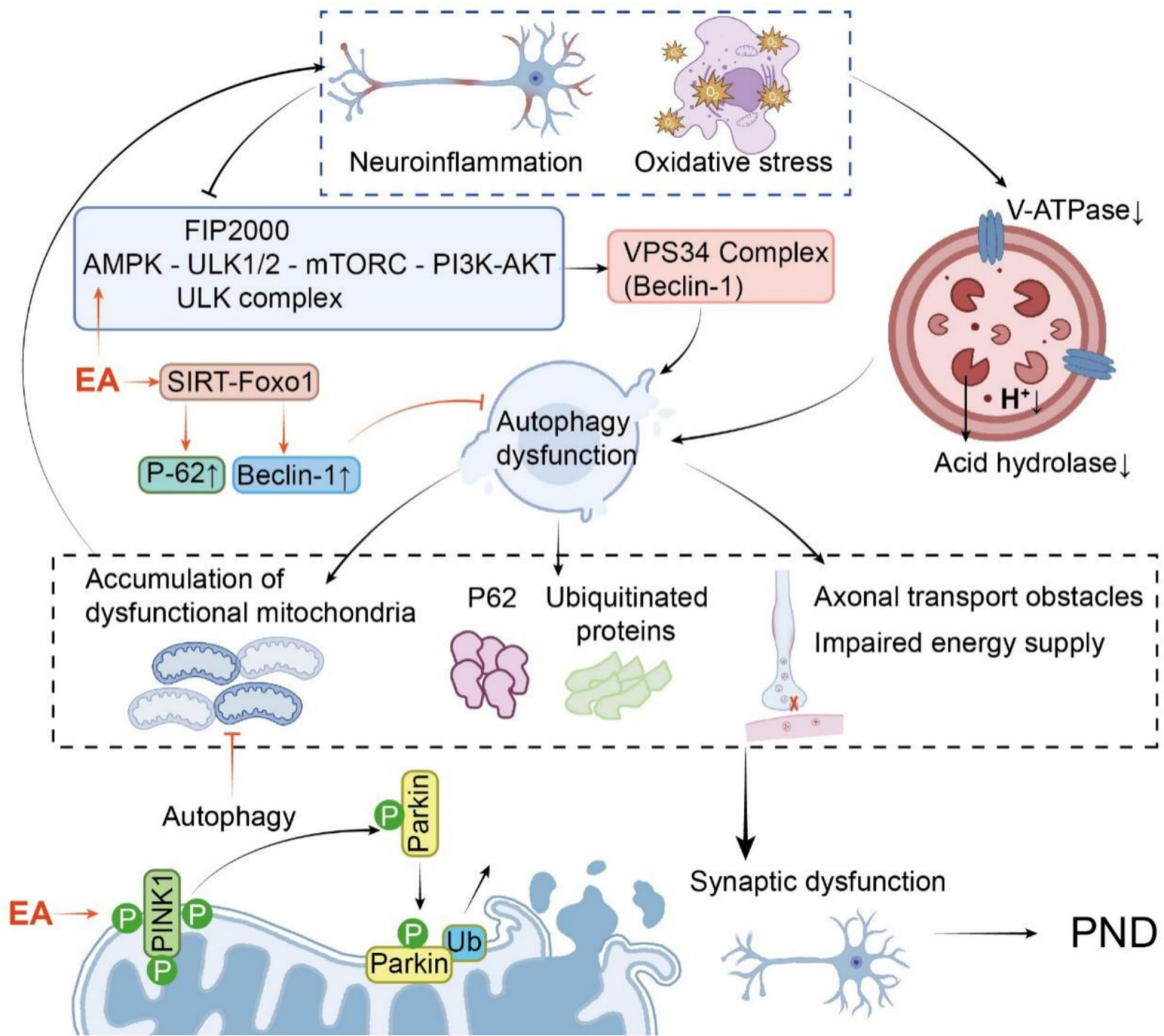
Mechanisms of autophagy dysfunction in PND and potential regulation by EA

However, the current evidence should be interpreted cautiously. Reports on whether EA “promotes” or “inhibits” autophagy in PND are not fully consistent, partly because autophagy initiation and autophagic flux are often not distinguished clearly. Most EA-PND studies still rely on static markers, such as LC3-II, Beclin-1, p62, and PINK1/Parkin, rather than dynamic assessment of autophagic flux. Therefore, changes in these markers may not fully reflect the actual efficiency of autophagic degradation. Future studies should use dynamic monitoring approaches, such as tandem fluorescent mRFP-GFP-LC3 probes, lysosomal function assays, and mitophagy flux assessment, to determine whether EA primarily corrects excessive autophagy initiation, restores downstream lysosomal clearance, or coordinates both processes in PND models [113–115]. Such studies will help clarify the stage-dependent regulation of autophagy by EA and provide a stronger basis for autophagy-targeted interventions in PND (Fig. 3).

Neuroinflammation and oxidative stress may impair autophagic homeostasis in PND by disrupting autophagy initiation, autophagosome maturation, lysosomal acidification, and downstream autophagic flux. Dysfunctional autophagy can lead to the accumulation of damaged mitochondria, p62, ubiquitinated proteins, and other abnormal substrates, thereby aggravating oxidative stress, neuronal injury, axonal transport defects, impaired energy supply, synaptic dysfunction, and cognitive impairment. EA may modulate autophagy-related processes through several pathways, including AMPK/ULK1-related autophagy initiation, SIRT1/FoxO1-mediated autophagy regulation, Beclin-1-associated autophagosome formation, and PINK1/Parkin-mediated mitophagy. These effects may help restore mitochondrial quality control and neuronal homeostasis in PND. However, most current EA studies in PND rely on static autophagy markers such as LC3, Beclin-1, p62, and PINK1/Parkin, whereas direct dynamic assessment of autophagic flux remains limited. Therefore, EA-mediated regulation of autophagy should be interpreted as stage-dependent and requires further validation using dynamic flux-monitoring approaches.

### Synaptic plasticity, neural electrical activity, and brain network connectivity

The cellular and molecular abnormalities described above may ultimately converge at the level of synaptic plasticity, neural electrical activity, and large-scale brain network connectivity. In PND, neuroinflammation, oxidative stress, autophagy dysfunction, mitochondrial injury, and glial activation can impair neuronal survival and synaptic function [116–118]. At the synaptic level, these changes may disrupt long-term potentiation (LTP), a key cellular process involved in learning and memory [119]. At the network level, impaired synaptic plasticity may reduce the efficiency of neuronal communication, disturb synchronized firing across distributed brain regions, and contribute to abnormal electroencephalographic activity and impaired functional connectivity within higher-order cognitive networks such as the default mode network (DMN) [120]. These downstream neurophysiological changes may help explain deficits in memory, attention, and executive function after surgery and anesthesia [121].

PND-specific studies support the relevance of synaptic plasticity and BDNF-related signaling in postoperative cognitive impairment. Xue et al. showed that BDNF/proBDNF imbalance contributes to postoperative cognitive dysfunction in aged mice by modulating synaptic plasticity [122]. Wu et al. further reported that SIRT1/BDNF downregulation in the hippocampal CA1 region was associated with impaired synaptic plasticity and decreased glutamatergic neuronal excitability in postoperative cognitive dysfunction [123]. These studies did not test EA intervention, but they help define BDNF-related synaptic plasticity and neuronal excitability as disease-relevant targets in PND.

Against this disease background, Huang et al. provided direct EA-PND evidence linking EA with hippocampal synaptic plasticity-related signaling. In aged rats with anesthesia/surgery-induced cognitive decline, EA pretreatment activated the ERK/MAPK/CREB pathway in the hippocampal CA1 region and improved postoperative cognitive performance [124]. Because CREB is an activity-dependent transcription factor closely related to synaptic plasticity and neurotrophic signaling, this study suggests that EA may help preserve postoperative cognitive function by regulating hippocampal ERK/MAPK/CREB-mediated plasticity pathways. However, this evidence mainly supports a synaptic plasticity-related molecular mechanism; direct evidence that EA regulates EEG patterns, LTP dynamics, or large-scale brain network connectivity in PND models remains limited.

More recently, Cheng et al. provided additional direct EA-PND evidence linking EA with hippocampal synaptic plasticity. In aged rats with sevoflurane anesthesia plus exploratory laparotomy-induced POCD, EA pretreatment at PC6, LI4, and ST36 improved postoperative recognition memory, restored neuronal morphology in the hippocampal CA1 region, and increased synaptic plasticity-related markers, including PSD-95 and synaptophysin. Mechanistically, EA reduced p38 MAPK phosphorylation, while pharmacological activation of p38 MAPK partly weakened the protective effects of EA. These findings suggest that inhibition of p38 MAPK signaling may contribute to EA-mediated preservation of hippocampal synaptic plasticity in POCD. However, because this study mainly assessed molecular and structural synaptic markers rather than EEG activity or large-scale functional connectivity, it should be interpreted as direct evidence for synaptic plasticity-related regulation, rather than direct evidence for large-scale brain network modulation [125].

Evidence from related non-PND models provides additional but indirect support. In a 5xFAD mouse model of Alzheimer’s disease, 40 Hz EA increased the frequency and amplitude of hippocampal excitatory postsynaptic currents, suggesting improved synaptic transmission efficiency [126]. In a vascular cognitive impairment model, EA rescued LTP deficits in the hippocampal CA3–CA1 pathway and increased phosphorylation of synaptic proteins such as GluR1, NMDAR2B, and CaMKII [127]. Other studies in Alzheimer’s disease, mild cognitive impairment, vascular cognitive impairment, and healthy aging populations suggest that acupuncture or EA may modulate functional connectivity involving the hippocampus, prefrontal cortex, limbic system, neocortex, and DMN [128–131]. These findings support the biological plausibility that EA may influence neural activity and brain network integration, but they should be interpreted as indirect mechanistic support rather than definitive evidence in PND.

**Fig. 4:**
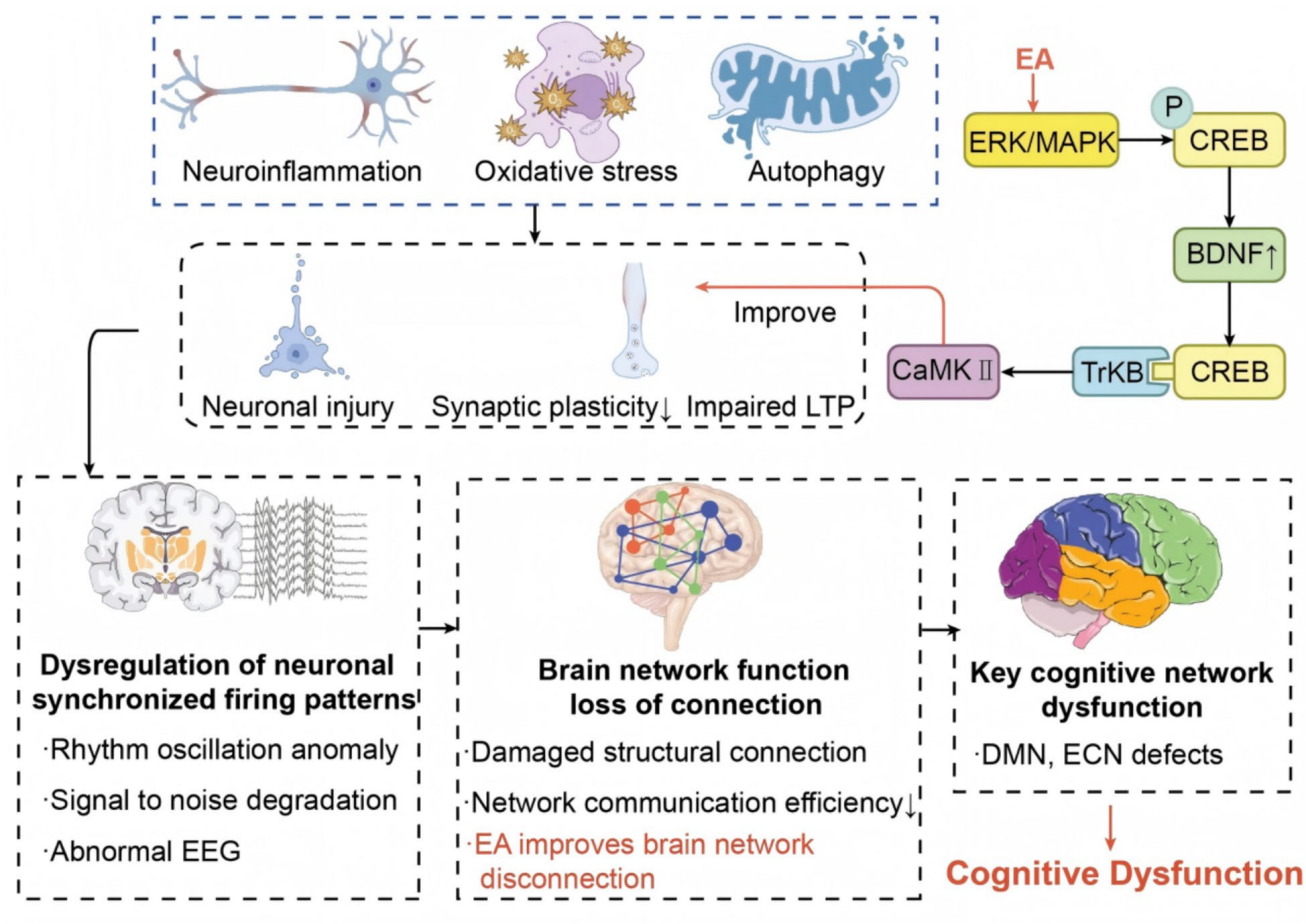
Synaptic and network dysfunction in PND and potential regulation by EA

Overall, current evidence suggests that neural electrical activity and brain network connectivity may represent important downstream manifestations of PND-related molecular and cellular injury. For EA, the most direct PND-model evidence in this domain currently centers on hippocampal MAPK-related synaptic plasticity regulation, including ERK/MAPK/CREB activation and p38 MAPK inhibition. In contrast, EA-mediated regulation of LTP, EEG oscillations, and large-scale functional connectivity in PND remains insufficiently validated. Future studies should combine postoperative animal models with behavioral testing, hippocampal electrophysiology, LTP recording, EEG analysis, and functional neuroimaging to determine whether the synaptic and network-level effects suggested by related disease models can be directly reproduced in the perioperative setting (Fig. 4).

Molecular and cellular abnormalities in PND, including neuroinflammation, oxidative stress, autophagy dysfunction, mitochondrial injury, glial activation, and neuronal damage, may converge on impaired synaptic plasticity and downstream network dysfunction. These changes may disrupt long-term potentiation, neuronal synchronization, electroencephalographic activity, brain network communication efficiency, and key cognitive networks such as the default mode network (DMN) and executive control network (ECN). Current EA-PND evidence most directly supports regulation of hippocampal synaptic plasticity-related signaling, including ERK/MAPK/CREB activation and p38 MAPK inhibition. These pathways may help preserve synaptic structure and plasticity-related proteins, such as PSD-95 and synaptophysin, thereby contributing to postoperative cognitive protection. However, direct evidence that EA regulates LTP dynamics, EEG oscillations, or large-scale functional connectivity in PND-specific models remains limited. Evidence for BDNF/TrkB-related plasticity and network-level modulation is still derived partly from related neurological disease models or non-postoperative populations, including Alzheimer’s disease, vascular cognitive impairment, mild cognitive impairment, and healthy aging. Therefore, these network-level mechanisms should be interpreted as indirect mechanistic support or biologically plausible hypotheses rather than definitively established EA mechanisms in PND. Further studies combining postoperative models with electrophysiology, EEG, and functional neuroimaging are needed.

### Additional mechanisms

In addition to neuroinflammation, oxidative stress, and autophagy dysfunction, apoptosis, ferroptosis, mitochondrial energy metabolism, and proteostasis-related dysfunction may also be involved in PND. However, the strength of evidence for EA-mediated regulation of these mechanisms is uneven. Therefore, this section focuses mainly on mechanisms with direct EA-PND evidence, while briefly discussing disease-context evidence and future research directions for pathways that have not yet been directly tested with EA in PND models.

Among these additional mechanisms, ferroptosis and mitochondrial bioenergetic dysfunction currently have relatively direct support from an EA-treated PND model. Du et al. used an aged mouse model of sevoflurane anesthesia plus tibial fracture surgery and showed that EA pretreatment improved cognitive impairment and inhibited ferroptosis through the SIRT1/NRF2/GPX4 pathway [132]. In the same study, EA increased ATP content, restored mitochondrial membrane potential, and improved mitochondrial ultrastructure, suggesting that EA may modulate ferroptosis-related cell death and mitochondrial energy metabolism in the perioperative setting. These findings provide direct PND-model evidence linking EA-mediated cognitive protection with ferroptosis suppression and mitochondrial bioenergetic homeostasis. Nevertheless, this evidence remains limited, and further validation in independent PND models is still needed.

Apoptosis is also relevant to perioperative neuronal injury, because mitochondrial damage can promote cytochrome c release, caspase activation, and neuronal apoptosis. However, direct EA-PND evidence specifically targeting the classical apoptotic pathway remains limited. Therefore, apoptosis is discussed here as a plausible mechanism related to mitochondrial injury and regulated cell death, rather than as a fully established EA-mediated pathway in PND.

Recent PND-specific single-cell evidence further supports the relevance of metabolic and proteostasis-related abnormalities in postoperative cognitive impairment. Suo et al. reported that postoperative neurons and glial cells showed protein dysmetabolism, mitochondrial abnormalities, impaired autophagy, and altered neuroglial communication [133]. These findings provide useful disease-context evidence that mitochondrial energy imbalance and protein metabolic dysregulation may contribute to PND. However, because this study did not test EA intervention, it should not be interpreted as direct evidence that EA regulates these pathways. Instead, it highlights potential targets for future EA studies.

Protein homeostasis and proteomics represent another important but still underexplored direction. The ubiquitin–proteasome system (UPS) is involved in protein quality control and may be affected by anesthesia and surgical stress, leading to abnormal protein accumulation, synaptic dysfunction, mitochondrial injury, and neuroinflammation [134, 135]. These processes may interact with ferroptosis-related pathways, such as GPX4 stability and lipid peroxidation. However, direct evidence showing that EA regulates UPS activity, protein turnover, or proteostasis-related neuroglial dysfunction in PND is still lacking. From an omics perspective, proteomic analyses may help determine whether EA induces changes in protein abundance, protein interaction networks, post-translational modifications, and pathway-level protein turnover. At present, proteomics should therefore be presented as a future research direction rather than an established mechanism of EA-mediated neuroprotection in PND.

Overall, current evidence suggests that EA may influence additional PND-related mechanisms, especially ferroptosis and mitochondrial energy metabolism. In contrast, apoptosis, UPS-related proteostasis, and proteomics-level regulation remain less directly supported in EA-treated PND models. Future studies should clarify whether EA directly regulates these pathways in PND-specific models and should combine behavioral testing with mitochondrial functional assays, cell death markers, and proteomic or multi-omics analyses.

## Clinical evidence for EA in PND

Although preclinical studies provide mechanistic support for EA in PND, clinical translation depends on evidence from randomized controlled trials, systematic reviews, and well-designed comparative studies. Current clinical evidence suggests that EA and related acupuncture-based electrical stimulation techniques may reduce early postoperative cognitive decline and improve short-term perioperative cognitive outcomes in older surgical patients. However, available studies differ substantially in intervention modality, stimulation parameters, acupoint selection, timing of intervention, surgical population, outcome assessment, and methodological quality. Therefore, the clinical evidence should be interpreted according to intervention type and protocol design rather than treating acupuncture-related interventions as a single uniform therapy.

Evidence from systematic reviews and meta-analyses supports the potential clinical value of perioperative EA, but also highlights important limitations. Ou et al. summarized randomized controlled trials in older adults undergoing hip or knee arthroplasty and reported that EA pretreatment reduced postoperative cognitive dysfunction at several postoperative time points, improved cognitive scores, and was associated with favorable changes in inflammatory and neuronal injury-related biomarkers [136]. More recently, Wu et al. conducted a systematic review and meta-analysis including 26 randomized controlled trials with 2,309 elderly patients undergoing general anesthesia. Perioperative EA was associated with reduced PND incidence, improved MMSE scores, decreased inflammatory biomarkers including IL-6, IL-1β, and TNF-α, reduced S100β levels, and showed a favorable safety profile [137]. However, the certainty of evidence varied across outcomes, and the authors emphasized methodological issues such as insufficient blinding, possible selection bias, high biomarker heterogeneity, and non-standardized EA protocols. These findings support EA as a promising perioperative intervention, but they do not yet establish an optimal EA regimen.

Recent randomized trials further suggest that the effect of EA-related interventions may depend on surgical context and intervention timing. Long et al. evaluated pre- and postoperative EA or TEAS (Transcutaneous Electrical Acupoint Stimulation) in elderly patients with hip fractures and reported lower PND incidence on postoperative days 1 and 3, accompanied by changes in IL-1β, IL-6, S100β, pain scores, and postoperative nausea and vomiting [138]. In geriatric patients undergoing gastrointestinal tumor surgery, Xi et al. found that perioperative TEAS, delivered from 30 min before anesthesia induction until the end of surgery and repeated before and after surgery, was associated with higher postoperative MMSE scores, lower POCD incidence, and reduced IL-6, S100β, and C-reactive protein levels [139]. Similarly, in older patients undergoing video-assisted thoracoscopic surgery, TEAS at PC6 and ST36 from 30 min before surgery until the end of surgery was associated with reduced early POCD incidence, improved MMSE and MoCA scores, and lower S100β and NSE levels [140]. These findings suggest that electrical acupoint stimulation may be feasible across different surgical populations, although the observed benefits are mainly concentrated in early postoperative cognitive outcomes.

Different acupuncture-related modalities should also be distinguished. EA involves needle insertion combined with electrical stimulation, whereas TEAS delivers electrical stimulation through surface electrodes without skin penetration. Manual acupuncture does not use electrical stimulation and may have different sensory, neurophysiological, and placebo-related features. Tu et al. investigated preemptive manual acupuncture in older patients undergoing hip replacement and reported improved postoperative MMSE and MoCA scores and lower early POCD incidence compared with sham acupuncture. These effects were accompanied by increased miR-124 and miR-146a levels and reduced TNF-α, IL-6, and IL-1β levels [141]. This trial suggests that manual acupuncture may also have perioperative cognitive benefits, possibly through anti-inflammatory regulation. However, direct head-to-head trials comparing EA with manual acupuncture for PND remain scarce. Therefore, EA, TEAS, and manual acupuncture should be discussed as related but not interchangeable interventions.

Comparative evidence from network meta-analysis further supports the need to distinguish acupuncture modalities. Liang et al. included 32 randomized controlled trials involving 2,644 elderly Chinese patients undergoing general anesthesia and compared several acupuncture-related interventions, including electroacupuncture, thumbtack needle, scalp acupuncture, auricular acupuncture, and Xingnao Kaiqiao acupuncture [142]. This analysis suggested that EA ranked highly for improving postoperative MMSE scores, while other modalities showed potential advantages in specific subgroups. However, because the included studies differed in surgical population, control design, acupoint prescription, stimulation protocol, and timing of intervention, these comparative rankings should be interpreted cautiously. Current evidence therefore supports modality-specific discussion but does not yet prove the superiority of one acupuncture modality over all others.

Acupoint selection varies across trials, but several core acupoints are repeatedly used. GV20 or DU20 and EX-HN1 are commonly selected for cognitive and central nervous system regulation; PC6 is frequently used for autonomic regulation, analgesia, and perioperative symptom control; ST36 and SP6 are often selected for systemic anti-inflammatory, immune-regulatory, and gastrointestinal effects; and LI4 is commonly used for analgesic and perioperative regulatory purposes. Common clinical combinations include GV20/DU20, PC6, ST36, SP6, LI4, and EX-HN1, with additional acupoints selected according to surgical type and intervention purpose. However, few trials directly compare different acupoint prescriptions. It therefore remains unclear whether clinical effects are driven by specific acupoints, acupoint combinations, electrical stimulation, or broader perioperative care effects.

Intervention timing and stimulation parameters are major sources of clinical heterogeneity. Some protocols use preoperative preconditioning, often beginning 30 min before anesthesia induction or surgery and continuing until the end of surgery. Other protocols include postoperative repeated stimulation for several days, while some combine preoperative, intraoperative, and postoperative treatment. For example, Xi et al. used TEAS before anesthesia induction, during surgery, 1 day before surgery, and from postoperative day 1 to day 3 [139]. Long et al. evaluated pre- and postoperative electrical acupoint stimulation in elderly hip fracture patients [138]. These designs suggest that timing and treatment course may be important variables influencing clinical efficacy. Preoperative stimulation may act as a preconditioning strategy, intraoperative stimulation may reduce surgery- or anesthesia-induced inflammatory responses, and postoperative repeated stimulation may support recovery by modulating pain, inflammation, sleep, and stress responses. At the same time, EA and TEAS protocols differ in electrical frequency, waveform, current intensity, stimulation duration, number of sessions, and total treatment course. Some clinical and experimental protocols use sparse-dense stimulation such as 2/15 Hz, whereas many clinical TEAS studies apply stimulation from 30 min before anesthesia or surgery until the end of the operation. However, not all trials fully report waveform, frequency, intensity, or treatment course, and there is still no consensus regarding the optimal timing window or stimulation “dose” for PND prevention. This limits reproducibility and makes dose–response analysis difficult. Future trials should report acupoint location, stimulation device, waveform, frequency, current intensity, treatment duration, timing relative to anesthesia and surgery, number of sessions, total course, practitioner training, and sham-control design.

Representative individual clinical studies and protocols are summarized in Table 2 to facilitate comparison of surgery type, intervention modality, acupoint selection, treatment regimen, stimulation pattern, and main outcomes. Meta-analyses and network meta-analyses are discussed in the text but are not included in the table because the included trials varied substantially in intervention protocols.

**Table 2:** Summary of clinical studies on acupuncture-related interventions for PND

References	Surgery type	Intervention	Acupoint	Treatment regimen	Stimulation pattern	Main outcomes
Long et al. [138]	Hip fracture surgery	EA/TEAS	GV20, PC6, ST36, SP6	Pre- and postoperative stimulation; 30 min per session	2/100 Hz dense-disperse wave; intensity adjusted according to patient tolerance	Lower PND incidence on postoperative days 1 and 3; changes in IL-1β, IL-6, and S100β; lower postoperative pain score and PONV
Xi et al. [139]	Radical resection of gastrointestinal tumors under general anesthesia	TEAS	PC6, GV29, ST36	From 30 min before anesthesia induction until the end of surgery; 1 day before surgery and from postoperative day 1 to day 3, 30 min once daily except on the day of surgery	2/100 Hz dilatational wave	Higher postoperative MMSE scores; lower POCD incidence; lower IL-6, S100β, and CRP levels
Wei et al. [140]	Video-assisted thoracoscopic lobectomy under general anesthesia	TEAS	Bilateral PC6 and ST36	Started 30 min before anesthesia induction and maintained until the end of surgery	Alternative dense-disperse frequency of 2/10 Hz; 6–15 mA according to tolerance	Lower early POCD incidence; improved MMSE and MoCA scores; lower S100β and NSE levels
Tu et al. [141]	Hip replacement	Preemptive manual acupuncture	DU20 and EX-HN1	Preoperative/preemptive acupuncture intervention	Manual acupuncture; no electrical stimulation	Higher postoperative MMSE and MoCA scores; lower early POCD incidence; increased miR-124 and miR-146a; lower TNF-α, IL-6, and IL-1β levels
Qian et al. [143]	Hip or knee joint replacement	TEAS combined with integrated perioperative nursing	LI4, TF4, LR3, SP6	Intervention across three stages: the day before surgery, the day of surgery before entering the operating room, and the postoperative week	2/100 Hz automatic shifting dense-disperse wave; 30 min; intensity adjusted to the maximum tolerated level	Improved delirium severity, cognitive impairment, anxiety, and depression; no significant reduction in SSD or POD incidence
Wei et al. [144]	Laparoscopic radical prostatectomy	EA	ST36 and GV20	Begins 30 min before anesthesia induction and lasts 25–30 min	Dilatational wave at 2 Hz; sterile disposable needles connected to an electro-stimulator	Primary outcome: POD incidence during the first 3 postoperative days assessed by 3D-CAM; efficacy results pending

This table summarizes representative individual clinical studies and protocols discussed in the clinical evidence section. Systematic reviews, meta-analyses, and network meta-analyses are discussed in the text but are not included in this table because the intervention protocols varied substantially across included trials. The Wei et al. study is a protocol-level study; therefore, efficacy results are pending. *EA* electroacupuncture, *TEAS* transcutaneous electrical acupoint stimulation, *PND* perioperative neurocognitive disorder, *POCD* postoperative cognitive dysfunction, *POD* postoperative delirium, *PONV* postoperative nausea and vomiting, *MMSE* Mini-Mental State Examination, *MoCA* Montreal Cognitive Assessment, *CRP* C-reactive protein, *NSE* neuron-specific enolase, *SSD* subsyndromal delirium, *3D-CAM* 3-Minute Diagnostic Confusion Assessment Method

Several limitations should be considered when interpreting the current clinical evidence. First, many trials remain single-center studies with small or moderate sample sizes, and a large proportion were conducted in China, which may limit generalizability. Second, blinding and sham-control design remain difficult, especially when comparing invasive EA, non-invasive TEAS, manual acupuncture, and usual care. Third, most studies rely on MMSE or MoCA, while delirium-specific tools and detailed neuropsychological batteries are less consistently used. Fourth, some studies tested combined interventions, which makes it difficult to isolate the specific effect of acupoint stimulation. For example, Qian et al. reported that TEAS combined with an integrated perioperative nursing program improved delirium severity, cognitive impairment, anxiety, and depression after joint replacement, but did not significantly reduce SSD or POD incidence [143]. This suggests that combined non-pharmacological programs may be clinically useful, but their specific active components require further clarification.

Protocol-level studies indicate that future trials are moving toward more rigorous designs, but they cannot substitute for completed efficacy trials. Wei et al. described a double-center, randomized, patient- and assessor-blinded, sham-controlled trial designed to evaluate EA for postoperative delirium in elderly patients undergoing laparoscopic radical prostatectomy. The protocol uses ST36 and GV20, begins 30 min before anesthesia induction, maintains stimulation for 25–30 min, and uses the 3D-CAM to assess POD during the first three postoperative days [144]. Such protocols may help address current limitations by using predefined acupoints, standardized timing, sham control, and delirium-specific assessment. However, until trial results are available, this should be regarded as evidence of ongoing research direction rather than evidence of clinical efficacy.

Overall, current clinical evidence suggests that EA and related electrical acupoint stimulation interventions may reduce early postoperative cognitive decline and improve short-term perioperative cognitive outcomes in older surgical patients. The evidence is relatively more consistent for early postoperative cognitive scores and inflammatory or neuronal injury-related biomarkers, whereas evidence remains limited for long-term cognitive protection, delirium-specific outcomes, optimal stimulation parameters, and comparative efficacy among EA, TEAS, and manual acupuncture. Future multicenter, adequately powered, sham-controlled trials should use standardized acupuncture reporting, longer follow-up, comprehensive neuropsychological and delirium assessments, and mechanistic biomarkers to determine the optimal modality, timing, acupoint prescription, and stimulation protocol for PND prevention and management.

## Conclusion

PND presents a major clinical challenge in an aging surgical population. This review summarizes current mechanistic and clinical evidence suggesting that EA may act on several interacting pathological processes, including neuroinflammation, oxidative stress, autophagy dysfunction, regulated cell death, mitochondrial energy metabolism, synaptic plasticity, and brain network regulation. These mechanisms are not isolated. Perioperative inflammation, oxidative injury, mitochondrial dysfunction, impaired autophagy, and glial activation may reinforce one another and ultimately converge on neuronal injury, synaptic impairment, altered neural activity, and cognitive dysfunction. EA may have potential value as an adjunctive non-pharmacological intervention because of its multimodal regulatory properties. However, the strength of evidence differs across mechanisms, and several proposed pathways remain supported mainly by indirect evidence from related disease models rather than by direct validation in EA-treated PND models.

Animal studies provide useful mechanistic clues, particularly for EA-mediated regulation of neuroinflammation, oxidative stress, autophagy, ferroptosis, mitochondrial function, and hippocampal synaptic plasticity. Recent PND-specific animal studies have strengthened the direct evidence base by linking EA with pathways such as NLRP3 inflammasome regulation, cGAS–STING signaling, microbiota–gut–brain axis modulation, ferroptosis-related SIRT1/NRF2/GPX4 signaling, and MAPK-related synaptic plasticity. Nevertheless, direct evidence remains uneven. Some mechanisms, including BBB regulation, DAMPs–RAGE/HMGB1 signaling, large-scale brain network connectivity, classical apoptosis, UPS-related proteostasis, and proteomics-level regulation, still require further validation in PND-specific experimental settings. Therefore, future studies should clearly distinguish mechanisms directly tested in EA-treated PND models from hypotheses inferred from related neurological diseases.

At the clinical level, current evidence suggests that EA and related acupoint-based electrical stimulation techniques may reduce early postoperative cognitive decline and improve short-term cognitive outcomes in older surgical patients. However, the available clinical studies remain limited by small or moderate sample sizes, single-center designs, geographical concentration, incomplete blinding, heterogeneous acupoint prescriptions and stimulation parameters, and reliance on cognitive screening scales such as MMSE or MoCA. Evidence is relatively more consistent for early postoperative cognitive scores and inflammatory or neuronal injury-related biomarkers, whereas evidence remains limited for long-term cognitive protection, delirium-specific outcomes, optimal stimulation protocols, and comparative efficacy among EA, TEAS, and manual acupuncture.

Another important gap is the lack of direct protein-level and proteomics-based evidence for EA-mediated neuroprotection in PND. Most current studies focus on selected signaling pathways or a limited number of molecular markers. Systematic characterization of protein abundance, protein interaction networks, post-translational modifications, and protein turnover remains insufficient. Given that protein homeostasis and mitochondrial energy metabolism may contribute to PND pathogenesis, future studies should consider integrating proteomics with transcriptomics, metabolomics, cell-type-specific analyses, and behavioral outcomes to clarify how EA affects molecular networks over time and across brain regions.

Overall, EA appears to be a promising but not yet definitively established non-pharmacological strategy for PND prevention and management. Future research should prioritize clinically relevant PND animal models, standardized EA protocols, dynamic mechanistic assessments, and multicenter, adequately powered, sham-controlled randomized trials. Such studies should combine comprehensive neuropsychological testing, delirium-specific assessment, long-term follow-up, and objective biomarkers. Only with this more rigorous evidence base can the relationship between EA mechanisms and clinical efficacy be clarified and its role in perioperative cognitive care be more precisely defined.

## Data Availability

No datasets were generated or analysed during the current study.

## Abbreviations

AD: Alzheimer’s disease
ALFF: Amplitude of low-frequency fluctuations
AMPK: AMP-activated protein kinase
ASC: Apoptosis-associated speck-like protein containing a CARD
BBB: Blood–brain barrier
BDNF: Brain-derived neurotrophic factor
CaMKII: Ca^2^⁺/calmodulin-dependent protein kinase II
cGAS: Cyclic GMP–AMP synthase
CNS: Central nervous system
CREB: CAMP response element-binding protein
DAMPs: Damage-associated molecular patterns
DMN: Default mode network
DMT1: Divalent metal transporter 1
dNCR: Delayed neurocognitive recovery
EA: Electroacupuncture
ECN: Executive control network
EEG: Electroencephalography
EPSCs: Excitatory postsynaptic currents
fMRI: Functional magnetic resonance imaging
FOXO1: Forkhead box protein O1
FPN: Ferroportin
GPX4: Glutathione peroxidase 4
HMGB1: High mobility group box 1
IL-1β: Interleukin-1 beta
IL-6: Interleukin-6
IRF3: Interferon regulatory factor 3
LC3: Microtubule-associated protein 1 light chain 3
LPS: Lipopolysaccharide
LTP: Long-term potentiation
MAPK: Mitogen-activated protein kinase
MCs: Mast cells
MCI: Mild cognitive impairment
MMSE: Mini-Mental State Examination
MoCA: Montreal Cognitive Assessment
MMPs: Matrix metalloproteinases
mPTP: Mitochondrial permeability transition pore
mtDNA: Mitochondrial DNA
mTOR: Mammalian target of rapamycin
NF-κB: Nuclear factor kappa B
NLRP3: NOD-like receptor family pyrin domain-containing 3
Nrf2: Nuclear factor erythroid 2-related factor 2
NSE: Neuron-specific enolase
PD: Parkinson’s disease
PI3K: Phosphatidylinositol 3-kinase
PINK1: PTEN-induced kinase 1
PND: Perioperative neurocognitive disorders
POCD: Postoperative cognitive dysfunction
POD: Postoperative delirium
PONV: Postoperative nausea and vomiting
RAGE: Receptor for advanced glycation end products
RCTs: Randomized controlled trials
RNS: Reactive nitrogen species
ROS: Reactive oxygen species
SIRT1: Sirtuin 1
SSD: Subsyndromal delirium
STING: Stimulator of interferon genes
TEAS: Transcutaneous electrical acupoint stimulation
TERT: Telomerase reverse transcriptase
TFR1: Transferrin receptor 1
TLR: Toll-like receptor
TNF-α: Tumor necrosis factor alpha
TrkB: Tropomyosin receptor kinase B
UPS: Ubiquitin–proteasome system
VCI: Vascular cognitive impairment
